# China's ‘sky eye’: exploring the universe by measuring faint cosmic radio signals

**DOI:** 10.1093/nsr/nwad003

**Published:** 2023-01-05

**Authors:** Weijie Zhao

**Affiliations:** Weijie Zhao is an NSR news editor based in Beijing

The Five-Hundred-Meter Aperture Spherical Radio Telescope (FAST) is the world's largest and most sensitive filled-aperture single-dish radio telescope, with a dish diameter of 500 meters and an effective illuminated diameter of 300 meters. This ‘big eye looking into the sky’ is located in a natural karst rock basin in Guizhou Province in China, where the human-related radio background is rather low, so it can better listen to radio signals coming from outer space. The construction of the FAST began in 2011, and the first light was observed in 2016. In 2021, it was officially opened to the global scientific community. According to FAST Chief Scientist Prof. Di Li of the National Astronomical Observatories (NAO) of the Chinese Academy of Sciences (CAS), ∼400 projects have been approved and 10% of the total observing time now goes to international scientists.

Before the construction of the FAST, the Arecibo Telescope of the USA (which terminated its function in 2020) was the largest filled-aperture radio telescope. The overall designs of these two telescopes are similar, with one significant difference—the dish of the Arecibo was essentially fixed, whereas that of the FAST is an ‘active surface’ consisting of ∼4500 metal panels so that the shape can be slightly altered to better focus the radio signals.

Why are scientists interested in catching faint radio signals from the universe? What can these signals tell us? The answers lie in three major astronomical areas: to identify and characterize pulsars, to understand the nature of fast radio bursts and to locate the neutral hydrogen gas in and out of our galaxy. Started in 2020, the Commensal Radio Astronomy FAST Survey (CRAFTS) project led by Prof. Di Li aims to simultaneously survey all three types of signals, covering the visible sky of the FAST within five years. Besides CRAFTS, there are four other ongoing FAST key science projects focusing on these scientific goals from different angles.

## DISCOVERING NEW PULSARS

A pulsar is a highly magnetized rotating neutron star that emits electromagnetic beams out of its two magnetic poles. Observers on Earth can catch periodical signals from it when its emissions are pointing toward Earth. Previously, ∼3000 pulsars have been discovered, and 700 more have been discovered in the past three years by FAST projects. CRAFTS discovered more than 160 new pulsars, and the Galactic Plane Pulsar Snapshot (GPPS) survey led by Prof. JinLin Han of NAO identified more than 500 new pulsars (it is estimated that up to 1000 will be discovered before the project's completion). ‘Particularly interesting are more than 120 pulsars with a period of a few milliseconds. Their properties are waiting to be revealed by observations,’ said Han.

Many of the pulsars newly discovered by the FAST in our galaxy are much fainter—generally 10 times fainter than previously identified pulsars. ‘Also, some pulsars discovered by FAST are much farther away, and they have been used as probes to reveal the magnetic fields in interstellar space in a much larger region than previously known,’ said Han. Some results of their study on interstellar magnetic fields were published recently in a special topic of *Science China Physics, Mechanics & Astronomy*.

‘Pulsars have extreme states of matter inside the neutron star and surprising plasma physics in the magnetosphere, which have all not been understood yet,’ said Han, ‘We know pulsars are shining in the sky, but do not know how they shine. We know there are pulsars inside the Milky Way, but do not know how many. The GPPS survey can help us to understand the population, distribution and properties of the pulsars in our Milky Way.’

## PROBING GRAVITATIONAL WAVES WITH PULSAR TIMING ARRAYS

Interestingly, the measurement of the time of arrival of electromagnetic pulses generated by pulsars, or *pulsar timing*, could provide a missing but significant piece of information for gravitational wave astronomy. Gravitational waves disturb the space-time between the pulsars and the Earth, thus can alter the observed steady pulsar periods. In particular, a pulsar timing array (PTA), which means the timing observations of an array of pulsars for a couple of years or even decades, has the ability to detect gravitational waves in the nanoHertz band. ‘In such a band, we aim at observing gravitational waves from supermassive blackhole binaries (SMBHs), the most massive celestial objects known so far,’ says Prof. Kejia Li of Peking University. ‘The expected SMBH population for PTA detection is located much further away from us than stellar mass blackholes, the target of ground-based laser interferometers such as Laser Interferometer Gravitational-Wave Observatory (LIGO). Thus, PTA really opens the observation window towards the deep universe.’

**Figure:**
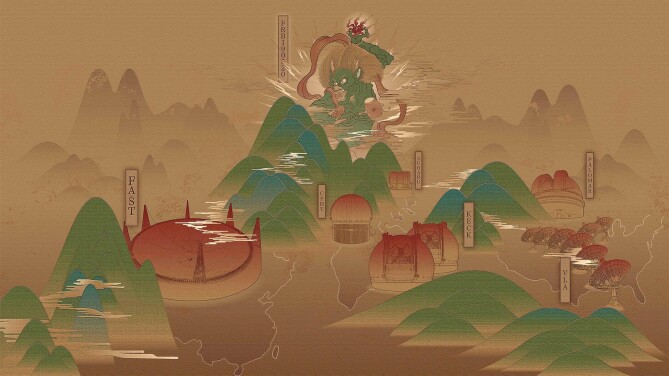
An artistic illustration: a repeating fast radio burst (FRB 20190520B) was associated with a persistent radio source by observations of FAST and other telescopes. (*Courtesy of Prof. Di Li and the CRAFTS project.*)

Prof. Kejia Li leads another FAST key scientific project that aims to build the Chinese pulsar timing array (CPTA). As the largest radio telescope in the world, the FAST can deliver the best pulsar timing precision for the L-band (1.0–1.5 GHz), which is a factor of 5 to 50 better than the current international pulsar timing array (IPTA) data set. ‘So far, CPTA has monitored more than 60 pulsars regularly for two years,’ Li said. ‘The discussion is open now for joining IPTA efforts and/or acting as an independent team to check and verify the IPTA findings.’

Detecting gravitational waves is not the only goal of CPTA. The PTA is also capable of observing the expected cosmic strings, and together with cosmic microwave background observations, helps to constrain quantum fluctuation in the early universe as well as phase transition processes. ‘This means the PTA is potentially capable of paving the way towards experimentation with regard to quantum gravity, one of the holy grails of fundamental physics,’ said Li.

## UNDERSTANDING THE NATURE OF FAST RADIO BURSTS

Since the commencement of the FAST’s operation, FAST data have facilitated more than 150 peer-reviewed journal articles, including seven *Nature* papers and one *Science* paper. Among these eight articles, six focused on FRBs and the other two focused on neutral hydrogen.

FRBs are bright and millisecond-duration astronomical radio pulses that were first discovered in 2007, and have been a hot and fast-developing research frontier in the past decade. To date, scientists have discovered more than 1000 FRBs but have not yet clarified their sources except for one specific FRB. ‘Because of the short-time-scale nature of FRBs, they are likely to be associated with compact objects like neutron stars or blackholes,’ explained NAO Prof. Weiwei Zhu, the leader of the FAST FRB key science project team. There are currently two general categories of models for the radiation mechanisms of FRBs: radiations from the magnetosphere of a compact object, and radiations from the relativistic shocks around compact objects.

‘So far, the evidence uncovered by FAST and other telescopes favors the magnetosphere of a magnetar as the radiation model for repeating FRBs. There is also increasing evidence of a varying and strongly magnetized unusual environment around the FRB source,’ said Zhu. In a 2020 article (*Nature* 2020; 587: 63), FAST data put the most stringent limit on radio bursts during the X-ray active phase of the FRB-emitting galactic magnetar, SGR J1935+2154. Together with the observation data of the Canadian Hydrogen Intensity Mapping Experiment (CHIME), the USA’s STARE-2 telescope and China's Hard X-ray Modulation Telescope, it was the first time that a magnetar had been distinguished as an FRB source.

However, there is also a population of FRBs that are never observed to repeat. ‘We call them the “one-off” FRBs,’ said Zhu. ‘They could be the result of some catastrophic event that is more extreme than magnetars. Whether or not these one-off FRBs are truly one-time events and have a different origin is still being debated.’ In the future, the discovery of more distant FRBs may help us to understand the origin of the one-off FRBs and apply FRBs to other astrophysical and cosmological problems.

The FAST FRB key science project team is diligently working on observing known repeating FRBs and searching for potential FRB host sources. Zhu said: ‘I think the mechanism for at least some of the FRBs could be established in the next few years.’

## MAPPING NEUTRAL HYDROGEN GAS

Another major scientific goal of the FAST is to map neutral hydrogen (HI) gas in and out of our galaxy by measuring the HI 21 cm transition signal that can be detected by radio telescopes. With the 19 beam receivers mounted, the FAST is one of the most powerful telescopes for this task.

‘HI 21 cm transition is humanity's main probe into the gaseous component of cosmic matter,’ explained Prof. Di Li. ‘The total mass of HI gas outweighs that of all the stars in all galaxies combined. Therefore, imaging and characterizing the galactic and extragalactic HI are fundamental tasks for astronomy.’ CRAFTS is producing high-quality HI images with 1% calibration accuracy, many times better than any of the existing large-scale surveys, and if endowed with necessary telescope time, it will produce the best HI images for the next two or three decades until the Square Kilometer Array (SKA) is constructed.

Within the Milky Way, mapping HI gas helps us to picture the structure of the galaxy. Outside the galaxy, mapping the intergalactic HI gas, which is the main component of intergalactic medium and the main material used to form stars and galaxies, is very helpful to understand the complicated physical process of the formation and evolution of galaxies. The FAST key project led by NAO Prof. Jie Wang aims to map the HI gas around the galaxy M31 (Andromeda) and its dark matter halo. M31 is located ∼2300 light years away from the Milky Way, and is the nearest typical galaxy.

‘If we want to have a complete census of the HI clouds around a galaxy or between galaxies,’ said Jie Wang, ‘for the Milky Way, we need to map the whole sky to get the complete view, which needs several radio telescopes lying in different regions around the world and is a big challenge; while for M31, the region of HI clouds around it is only covering less than 2% of the sky, and it makes the census easy.’ Through the FAST survey of M31, Wang and his colleagues will achieve the deepest HI mapping around a typical galaxy ever carried out. This will be very helpful for understanding the complicated physical processes that happen in a galaxy: tidal stripping, ram pressure, cold flow and jet eruption.

Furthermore, the pulsars that have been detected now are all in or around the Milky Way. ‘With the strong detection ability of the FAST, we have a unique chance to detect pulsars in another typical galaxy, the M31,’ said Wang.

The FAST has been one of the iconic big science facilities of China in recent years. It has and will continue to facilitate significant scientific discoveries, but its construction cost is actually not high—by 2016, when it observed the first light, the cost was as low as ∼1.15 billion yuan, which is only enough to build two subway stations in a big city.

In the future, the FAST will be further upgraded with a state-of-the-art phased-array-feed receiver to improve the survey efficiency by at least one order of magnitude, as the survey speed is currently the main bottleneck of its science output. Moreover, a FAST array will be built. ‘FAST, originally proposed by Dr. Rendong Nan and his colleagues as an SKA concept, consisted of 10–20 giant antennae,’ said Prof. Di Li. ‘To realize such a FAST array, with unparalleled depth of radio vision, will be a dream for Chinese radio astronomers of this generation and beyond.’

